# Tau Phosphorylation by GSK3 in Different Conditions

**DOI:** 10.1155/2012/578373

**Published:** 2012-05-17

**Authors:** Jesús Avila, Gonzalo León-Espinosa, Esther García, Vega García-Escudero, Félix Hernández, Javier DeFelipe

**Affiliations:** ^1^Centro de Biologia Molecular “Severo Ochoa” (CSIC-UAM), Nicolás Cabrera 1, Campus Cantoblanco UAM, 28049 Madrid, Spain; ^2^Centro de Investigación Biomédica en Red de Enfermedades neurodegenerativas (CIBERNED), 28031 Madrid, Spain; ^3^Instituto Cajal, Consejo Superior de Investigaciones Científicas, 28002 Madrid, Spain; ^4^Laboratorio Cajal de Circuitos Corticales, Centro de Tecnología Biomédica, Universidad Politécnica de Madrid, 28223 Madrid, Spain

## Abstract

Almost a 20% of the residues of tau protein are phosphorylatable amino acids: serine, threonine, and tyrosine. In this paper we comment on the consequences for tau of being a phosphoprotein. We will focus on serine/threonine phosphorylation. It will be discussed that, depending on the modified residue in tau molecule, phosphorylation could be protective, in processes like hibernation, or toxic like in development of those diseases known as tauopathies, which are characterized by an hyperphosphorylation and aggregation of tau.

## 1. Introduction

Tau protein was discovered as one of the brain microtubule-associated proteins bound to in vitro assembled tubulin [1]. Tau protein appears as a series of different polypeptides on gel electrophoresis. These different forms are generated by alternative RNA splicing [2] or by different phosphorylation levels [3]. In every tau form, four different regions could be identified: the amino terminal region, the proline-rich region, the microtubule binding region, and the carboxy terminal region. Here, we will mainly comment on the tau molecule of the largest tau form present in the central nervous system (CNS) [4]. This molecule contains 441 residues. The N-terminal region (1–150 residues) and the proline-rich region (151–239 residues) have been involved in the interaction of tau protein with cell membranes [5, 6]. The microtubule-binding region (residues 240–367) contains four similar but not identical sequences involved in the interaction with tubulin [7], the main component of microtubules. The last part of the molecule is the C-terminal region (residues 368 to 441). Curiously, for a specific residue, threonine, there is a decreasing gradient in its presence from the N-terminal to the C-terminal region in human tau. Threonine is particularly abundant at the N-terminal region [7]. Some of these threonines, residues 17, 30, 39, 50, 52, or 95, present in human tau are not always conserved in other species and may have appeared during evolution in human tau. None of these human tau threonines have been found to be phosphorylated, but we cannot rule out that some of these sites could be modified with a very fast turnover.

## 2. Tau Functions

Tau is a neuronal microtubule-associated protein, and some of its functions are related to that association that may result in microtubule stabilization or the regulation of axonal transport, but tau protein is a “sticky” protein that could bind to other proteins, apart from tubulin (the main component of microtubules), which could also facilitate its subcellular localization at the cytoplasm close to the membrane or in the nucleus. On the other hand, in the neuron, tau is mainly present in axons, owing to a sorting mechanism in which its level of phosphorylation may play a role [8], but tau can be also localized, at a low level, to dendrites [9] where it binds to PSD95 protein, a postsynaptic component present in dendritic spines, which is bound to glutamate (NMDA) receptors [10].

Some of tau functions could be regulated by phosphorylation. In many cases, tau phosphorylation can cause its detachment from microtubules [11], and those functions like microtubule stabilization and regulation of axonal transport could be affected by the change in the association of tau to microtubules.

## 3. Phosphotau

Tau is a phosphoprotein that could be modified by different protein kinases at tyrosine, threonine, or serine residues. In the longest CNS tau molecule, there are 80 serine/threonine residues that are mainly present at the proline-rich and C-terminal regions [11].

Tau kinases have been divided in proline- (PDPK) and nonproline- (NPDPK) directed protein kinases, being GSK3 the PDPK that could modify more sites in tau molecule [12]. These sites are also mainly present at the proline-rich and C-terminal regions.

GSK3 is a protein kinase originally identified due to its role in glycogen metabolism regulation. GSK3*β*, one of its isoforms, is abundant in the central nervous system and can modify several neuronal proteins like tau [13].

Tau hyperphosphorylation takes place, at the CNS, in several diseases (tauopathies), being the most predominant of these disorders, Alzheimer disease. In the tauopathies, tau protein is not only phosphorylated but also could be found in aggregated form. In Alzheimer's disease (AD) tau protein can be assembled into the paired helical filaments that form the aberrant structure known as neurofibrillary tangles [11]. Also, in AD, there is another aberrant structure the senile plaques, composed by the beta-amyloid peptide. A relationship between NFT and SP has been suggested through GSK3 (see below and [14]).

## 4. GSK3 and AD

In Alzheimer's disease postmortem brain material, tau phosphorylation has been found to increase at several sites modified by GSK3. In this way, the analysis of how an increase in GSK3 activity could modify tau protein has been deeply studied. It is known the involvement of factors like wnt, insulin, or insulin-like growth factor in the regulation of GSK3 [15]. It is also known that the accumulation of beta-amyloid peptide facilitates tau phosphorylation by GSK3 through the interference with insulin or wnt pathways [16, 17]. Also, mutations in the protein presenilin-1 (PS-1) that could result in the appearance of dementia with familiar origin may affect the interaction of PS-1 with the regulatory subunit of PI3 kinase, decreasing the activity of this kinase and the further activation of GSK3 [18]. On the other hand, in nonfamilial dementia like sporadic Alzheimer disease, the presence of apolipoprotein E4 isoform could facilitate a higher GSK3 activity [19]. Also, a GSK3 polymorphism has been associated with Alzheimer's disease [20], and an increase in GSK3 activity has been suggested to take place in the brain of AD patients [21]. Finally, GSK3 phosphorylates the majority of sites, on tau molecule, which are abnormally phosphorylated in AD [12].

One of the consequences of tau phosphorylation by GSK3 is a decrease of its interaction with microtubules [22], which could result in a destabilization of microtubule network [23]. Also, phosphorylation of tau by GSK3 (or other kinases) may result in an increase or a decrease in its interaction with other proteins. An increase was observed in its interaction with prolyl isomerase 1 (PIN-1), a chaperone protein that binds to phosphotau [24]. In this interaction phosphothreonine 231 is involved. On the other hand, Pin-1 could inhibit GSK3*β*, and a consequence of that is a reduced phosphorylation of amyloid precursor protein (APP), another important protein in AD. In the absence of phosphorylation, the turnover of APP is increased [25]. Also, Pin-1 acts on T668P motif in APP to promote non-amyloidogenic APP processing [26]. About GSK3 and APP processing it should be indicated a report indicating that GSK3, mainly GSK3*α*, can regulate APP processing and the production of amyloid beta peptide [27].

 Going back to tau phosphorylation, it has been observed that the interaction of tau with cell membranes decreases upon its phosphorylation [6]. In this binding, phosphorylation of serine 202 may play a role. Thus, the balance of kinase and phosphatases acting on tau could be important for tau functions and dysfunctions.

## 5. Is Phosphotau Toxic or Protective in a Neuron?

Since tau phosphorylation may affect tau functions, it was suggested that phosphotau could be toxic. However, this point has been widely discussed and is commented if phosphorylated tau is toxic, protective, or none of the above [28].

Working on *Drosophila* as a model it was indicated that phosphotau is not toxic [29, 30]. Also, there are some works suggesting that tau toxicity could be dependent in an increase in its expression [31] or that the toxicity could be related to its fragmentation, yielding some specific peptides [32], or to its aggregation, but this point is also discussed, since some works suggest a protective role for large tau aggregates, although it may be insufficient in damaged neurons [33, 34]. More recently, it has been suggested a toxicity for tau oligomers [35]. A possible relation between tau phosphorylation and aggregation has been suggested. In a mouse model for temporal dementia, chronic lithium (a GSK3 inhibitor) treatment results in a decreased tau phosphorylation and aggregation [36]. In another study a similar result was found [37], being the site recognized by antibody PHF-1 one of the phosphosites with a higher decrease upon treatment with a GSK3 inhibitor. It may suggest that tau phosphorylation at some sites may facilitate tau aggregation. These results support the previous works indicating that large tau aggregates exert negligible neurotoxicity compared with soluble tau [38, 39].

A protective role for tau phosphorylation could be suggested when that modification could take place at threonine 181 [40] that could facilitate its binding to exosomes and the release of tau excess [41]. Also phosphorylation at serine 202 could prevent the tau proteolysis by calpain [42], or in some processes, like hibernation (see below), it could be also protective [43]. Although poorly understood, there is an endocrine regulation of hibernation, and a recent study suggests that central system neurons are protected from low-temperature cell death by certain endogenous substances such as adenosine, opioids, and histamine [44].

On the other hand, tau toxicity resulting from tau phosphorylation could affect to axonal transport, mitochondrial respiration, or cytoskeletal changes [45, 46]. Also, the presence of hyperphosphorylated tau in dendritic spines impairs synaptic activity [47], and tau phosphorylation by GSK3 in the hippocampus results in a toxic gain of function [48, 49].

A way to study the role of tau phosphorylation on cell toxicity is the use of pseudophosphorylated tau variants where a serine or threonine residue is replaced by glutamatic acid. Thus, it has been found that pseudophosphorylation at the site recognized by antibody AT8, but not at the site recognized by abPHF-1, prevents the interaction of tau with neuronal membranes [50, 51].

Pseudophosphorylation of tau in ten different sites of a single molecule (including those sites recognized by AT8, ab PHF-1, and phosphothreonine 231) results in a toxic action and the induction of apoptosis [52].

Combined pseudophosphorylation at threonine 212, threonine 231, and serine 262 causes neurodegeneration, mainly through the presence of pseudophosphorylation at threonine 212 [53]. A possible mechanism has been involved for this toxicity: abnormal tau phosphorylation can result in the sequestration of unmodified tau [54] or in that of other microtubule associated proteins like MAP1 or MAP2 [23], promoting a net microtubule disassembly that could affect neuron cytoskeleton and promote neurodegeneration. On the other hand, tau phosphorylated at Thr 231 is used as a marker for neurodegeneration. It may suggest that tau modified at that residue could be toxic [55].

All those results suggest that, depending on the modified site, a protective or toxic function may take place and that it could be due to different conformational changes of tau molecule. Although tau is an unstructured molecule, it has been suggested that, in some conditions, it could have a paperclip conformation. In this conformation, the C-terminal end of tau interacts with the microtubule-binding domain, whereas the N-terminal end could bind to the C-terminal region, adopting a paperclip folding [56]. Pseudophosphorylation at the AT8 site makes the N-terminal region away from the C-terminal. On the other hand, pseudophosphorylation at the PHF-1 site decreases the interaction of C-terminal region with microtubule binding domain [57]. Thus, phosphorylation at different sites of tau molecule could have different consequences for tau conformations and function. In this way, we will comment more on the phosphorylation of tau on those sites recognized by ab AT8 or ab PHF-1.

## 6. The Progression of Alzheimer and the Phosphorylation of Tau by GSK3 at Two Different Sites

At the level of degeneration of a single neuron, occurring in Alzheimer's disease, it was suggested that first takes place phosphorylation of tau molecule at some specific sites and afterwards there is a further phosphorylation and aggregation of tau protein. Thus, early tau phosphorylation can be used as an initial marker for neurodegeneration [58].

The phosphorylation of tau, by GSK3, at specific sites can be analyzed by the use of antibodies that specifically recognize some of those sites. In this way, analysis of phosphorylation at serine 202 or at serines 396–404 could be achieved by using the antibodies AT8 [59] or PHF-1 [60], respectively. The uses of these antibodies have indicated that different tau molecules could be modified at those different sites [61]. On the other hand, in mature neurons tau phosphorylated at the site recognized by PHF-1 is preferentially present in the axon, whereas tau modified at AT8 site is located in the somatodendritic compartment [61]. Temporal lobe sections, including the entorhinal region and hippocampus, were studied using the antibody AT8 in AD patients and controls, to follow the consequences of that phosphorylation at the level of a single neuron and its possible relation with tau aggregation. The analysis showed that tau was modified at the site recognized by ab AT8 much earlier than the appearance of aggregated (fibrillar) tau, suggesting that phosphorylation at the AT8 site represents an earlier change than tau aggregation [58]. More recently, by looking at specific neurons in the brain of Alzheimer's disease patients like those present in the CA3 region of the hippocampus, the sequence for cellular changes related to tau phosphorylation and the posterior formation of aggregates has been studied with tau antibody AT8. This antibody recognizes the first stages of tau phosphorylation (pretangle stages) [62], whereas the reaction with tau antibody PHF-1 could correlate further phosphorylation steps and the formation of tau aggregates (tangle stages) [63].

Tau phosphorylation (recognized by AT8 antibody) at regions like CA3 of the hippocampus has been correlated with regression of synaptic components and memory deficits [64]. Indeed, although in AD there is neuron death, it has been suggested that there also could be alive neurons undergoing atrophy [65]. This atrophy could be the consequence of a metabolic decline [66] (that could be a very early sign of AD). Indeed, there is a reduced metabolism in those regions of the brain that are later affected in AD patients, and this glucose hypometabolism may reflect a reduced synaptic activity. This reduced metabolism may induce hypothermia, decreasing the activity of phosphatases that could result in the net phosphorylation of GSK3 substrates like tau protein [66].

A main difference between neuron atrophy and neuron death is that in the first case the neuron remains alive and could be possible to reactivate it. In fact, we have recently observed that in patients with AD the accumulation of phosphotau in a pretangle state does not induce changes in the dendrites of pyramidal neurons, whereas the presence of tau aggregates forming intraneuronal neurofibrillary tangles gives rise to a progressive dendritic atrophy and the loss of dendritic spines (synaptic disconnection) (Merino-Serrais P et al. in preparation). Thus, the presence of phosphotau in a pretangle state in neurons of the human brain does not necessarily result in severe and irreversible effects. Thus, it is of great interest to search for possible physiological conditions in which neurons with a reduced metabolism and altered synaptic connectivity that may result in the lack of cognitive functions could be reactivated to regular functionally neurons. The group of Arendt et al. [67] answers to that question by showing that topor in hibernating animals shows a significant analogy with the pretangle stages of AD. Hibernation is a behavioral strategy used by some mammals to minimize energy expenditure under inhospitable environmental conditions. During hibernation overall metabolic rates are greatly reduced and neurons like those of the CA3 hippocampal regions have a reduced metabolism [68]. Moreover, tau phosphorylation also takes place in those neurons, at sites recognized by AT8 antibody, phosphorylation which is fully reversed in a few hours when these animals spontaneously interrupt torpor by an arousal leading the animals to euthermia for 24 h approximately [69]. Furthermore, it has been shown that there is a re-establishment of mossy fiber synapses in CA3 neurons after arousal [70]. Previous studies have shown that neuronal cell bodies, dendrites, and spines in hibernating ground squirrels retract on entry into torpor state. When they return to euthermia, there is a recovery of the arbor complexity and spine density [71].

Thus, if topor stage, in hibernation, is similar to the pretangle stages in AD and it could be reversed, we could ask if that reversion could also take place in AD. This question has pros and cons. One of the cons is that the primary structure of tau from hibernating animals could be different than that of a human being. Thus, tau from hibernating mammals have been sequenced [70], but no dramatic differences with human tau were found. Then, how can we increase metabolic rate in AD neurons? In a further analysis, tau protein from *Mesocricetus auratus* (another hibernating rodent) has been sequenced and characterized (Leon G et al. in preparation). The reason of that characterization has been to perform a first step to know if tau protein in hibernating animals, like *M. auratus*, could be taken as a single marker of the process or it may play a role in it. Thus, tau differences between this hibernating animal and nonhibernating organisms like the human being should be carried out. Our results have indicated the presence of several variations at the N-terminal half between *M. auratus* and human tau. Some of these variations have been also found in nonhibernating rodents. However, there are three variations, in that region, which are only present in the two hibernating rodents, *M. auratus* and *S. citellus*. In addition, there are another differences between *M. auratus* and human tau like the presence of 10 phosphorylatable residues present in human but not in *M. auratus* tau (Leon G et al. in preparation). Many of these phosphorylatable residues could be possible substrates for ck1, a protein kinase that has been related with circadian rhythm in nonhibernating organism [72, 73]. Although additional work should be done, our observations are compatible with a possible role of tau in the hibernation process and not being only found as a single marker.

It could be a possible relationship between hibernation/wake in hibernating mammals and sleep/wake in humans. Indeed, AD patients exhibit sleep/wake disorders [74]. However, sleep deprivation has been associated with GSK3 activation [75], whereas a net GSK3 phosphorylation takes place in hibernating mammals [76]. Nevertheless, modulators of sleep/wake cycle, like melatonin, have been suggested as possible therapeutical compounds for AD [77].

A more related behavior to that of hibernation could be anesthesia. Anesthesia leads to tau hyperphosphorylation through inhibition of phosphatase activity by hypothermia [78], a process that could be similar to that of hibernation. However, little is known about how is the recovery from phosphotau to unmodified tau after anesthesia. In addition, we do not know if tau phosphorylation is sometimes associated with the physiological conditions following hibernation or anesthesia or, like in AD, may play a toxic function [49]. Thus, if phosphotau could be toxic and it is difficult to active phosphatases for its dephosphorylation, the use of possible GSK3 inhibitors should be taken into consideration for the treatment of the disease.

Furthermore, we should look for better analysis to detect a decreased metabolic rate in damaged CA3 neurons in AD, and we should look for an answer about how to increase that metabolic rate in AD neurons. A probable common link among some of these disorders is the appearance of oxidative damage that results in neurodegeneration [79, 80].

A possible mechanism has been suggested to explain an energy depletion in AD. It involves that oxidative stress may lead to the formation of reactive carbonyl compounds [81] that appear to increase in AD patients. These compounds may decrease glucose metabolism yielding to energy depletion in neuronal cells [82]. Also, intracellular A*β*  may cause mitochondrial defects and ATP depletion.

In summary, since neuron atrophy in neurodegenerative diseases, like AD, has been correlated with tau phosphorylation by GSK3, it should be tested in the future if by decreasing that phosphorylation that atrophy could be reversed or not.
